# High-Resolution CT–Based Virtual Simulation of an Unruptured Sinus of Valsalva Aneurysm for Aortic Root Reimplantation: A Case Report

**DOI:** 10.70352/scrj.cr.25-0780

**Published:** 2026-05-14

**Authors:** Haruo Yamauchi, Masahiko Ando, Yasuhiro Hoshino, Hiroyuki Kaneko, Katsura Soma, Yasutaka Hirata, Minoru Ono

**Affiliations:** 1 Department of Cardiovascular Surgery, The University of Tokyo Hospital, Tokyo, Japan; 2 Department of Cardiovascular Medicine, The University of Tokyo Hospital, Tokyo, Japan; 3 Division of Cardiovascular Surgery, National Center for Child Health and Development, Tokyo, Japan

**Keywords:** case report, sinus of Valsalva aneurysm, three-dimensional, computer graphics, valve-sparing aortic root replacement

## Abstract

**INTRODUCTION:**

Valve-sparing aortic root replacement (VSRR) for sinus of Valsalva aneurysm (SVA) is rarely reported due to technical challenges. In this case report, high-resolution CT data were used for 3D computer graphics (3DCG) visualization and precise measurement of aortic root geometry, facilitating VSRR in a patient with a markedly disproportionate, unruptured SVA.

**CASE PRESENTATION:**

A 36-year-old female experienced heart failure at delivery. Echocardiography revealed an SVA involving a dilated noncoronary sinus (NCS) (43 mm) extending over the right atrium, a dilated sinotubular junction, and moderate aortic regurgitation (AR). To assess the feasibility of VSRR, electrocardiogram-gated CT data were analyzed in 2 ways. First, multiplanar reconstruction of CT images allowed measurement of the virtual basal ring area (566.6 mm^2^, corresponding to 26.9 mm), cusp angles (left-coronary, right-coronary, and noncoronary cusps: 110°, 117°, and 133°, respectively), and commissural heights (16.2, 6.3, and 19.6 mm at the non–left, left–right, and right–non intercusp commissures, respectively). Second, high-quality 3DCG images were generated to simulate the surgeon’s intraoperative view. VSRR using the reimplantation technique was performed. Under an aortic cross-clamp, the SVA wall was incised, leaving a 5-mm margin above the annulus. The subvalvular tissue beneath the SVA appeared thin and fragile. A 32-mm straight graft was plicated to 29 mm at the bottom for subvalvular fixation. For the NCS, additional running sutures were applied between the graft’s bottom skirt and the remnant SVA wall to secure hemostasis. Commissures were fixed at predicted heights based on CT measurements; one commissure required a 1-mm adjustment using the water test. Supravalvular aortic rims were then secured inside the graft with 4-0 monofilament running sutures. No additional cusp repair was necessary. The patient was weaned from cardiopulmonary bypass smoothly, and her postoperative course was uneventful. Transthoracic echocardiography at discharge showed mild AR, which remained stable after 1 year.

**CONCLUSIONS:**

This case demonstrates successful aortic root reimplantation for an unruptured SVA. CT-based aortic root visualization and measurement provided clear virtual images, facilitating surgical planning and increasing confidence in performing valve-sparing procedures for SVAs with unusual anatomy.

## Abbreviations

3DCG
: 3D computer graphics
AR
: aortic regurgitation
BSA
: body surface area
cCL
: cusp coaptation length
CH
: commissural height
ICD
: intercommissural distance
LCC
: left-coronary cusp
LCS
: left-coronary sinus
NCC
: noncoronary cusp
NCS
: noncoronary sinus
RCC
: right-coronary cusp
STJ
: sinotubular junction
SVA
: sinus of Valsalva aneurysm
VBR
: virtual basal ring
VSRR
: valve-sparing aortic root replacement

## INTRODUCTION

Conventional surgical repair of congenital SVA typically involves direct or patch closure of the aneurysmal orifice, with or without concomitant aortic valve repair. However, postoperative suture-line leakage or recurrent AR often necessitates reintervention, particularly in patients with concomitant ventricular septal defect and AR.^1–3)^ VSRR for SVA repair has been reported infrequently,^4–11)^ yet it offers an alternative approach. This technique addresses the entire aortic root structure, including the VBR and STJ, but it remains technically challenging due to the extreme disproportion of the root geometry and the fragile aneurysmal wall. Recently, 3DCG based on CT data have been applied to plan intracardiac repair for complex congenital heart diseases.^12)^ We also previously demonstrated that graft sizing and CH for VSRR can be accurately predicted using preoperative CT data.^13)^ In this report, we present a case in which 3DCG visualization and CT-based measurements of a complex aortic root geometry were used to evaluate the feasibility of aortic root reimplantation for an unruptured SVA from a surgeon’s perspective.

## CASE PRESENTATION

**Fig. 1:**
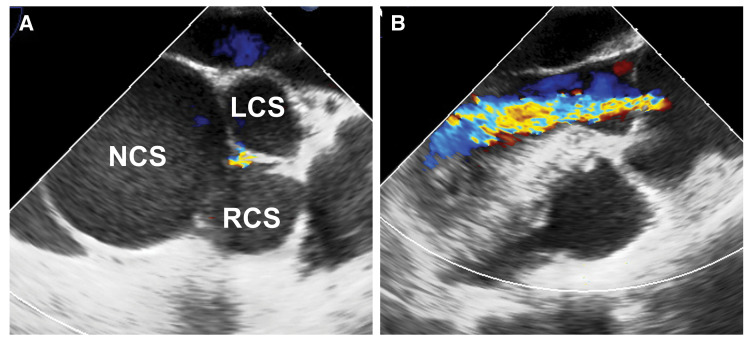
Preoperative transesophageal echocardiographic images. (**A**) Short-axis view of the aortic valve showing that the NCS is larger than the LCS and RCS. (**B**) Color Doppler long-axis view showing central moderate AR. AR, aortic regurgitation; LCS, left-coronary sinus; NCS, noncoronary sinus; RCS, right-coronary sinus

A 36-year-old female delivered a baby 3 years ago via cesarean section at 29 weeks of gestation due to fetal dysfunction. During the procedure, she experienced severe gestational hypertension and congestive heart failure. Transthoracic echocardiography revealed an SVA arising from a dilated NCS measuring 43 × 39 mm (**Fig. 1A**). The STJ and ascending aorta were also dilated to 39 and 44 mm, respectively. Color Doppler imaging demonstrated central moderate AR (**Fig. 1B**). A perimembranous ventricular septal defect was suspected due to a bulging membranous septum, although no shunt flow was detected. Surgical repair limited to closure of the SVA orifice would likely not correct the AR; thus, aortic root replacement was indicated. Given her comorbid obsessive-compulsive disorder and difficulty adhering to medication, she preferred to avoid lifelong anticoagulation. At the time of surgery, her height was 166 cm, her weight was 58.2 kg, and her BSA was 1.6 m^2^.

**Fig. 2:**
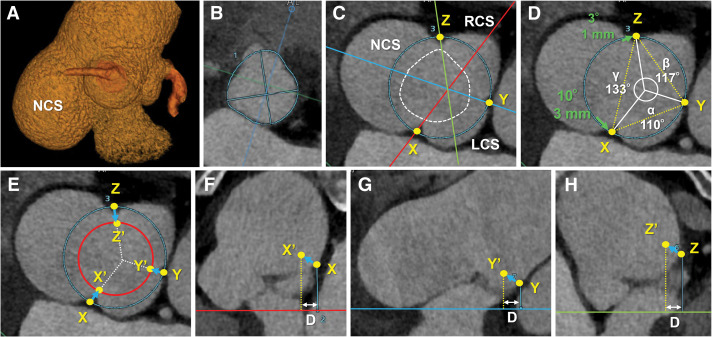
Preoperative CT assessment of the aortic root. (**A**) 3D CT images showing the SVA located at the NCS. (**B**) Multiplanar reconstruction at the VBR plane through the 3 nadir points of the LCC, RCC, and NCC. The blue curved line traces the VBR for area and perimeter calculation. (**C**–**E**) Multiplanar reconstructions at the commissural plane. The blue circle represents the commissural circle. Yellow points X, Y, and Z indicate commissural positions. (**C**) White dotted line overlaps the VBR. Red, blue, and green lines represent cutting planes connecting the center of the aortic valve (coaptation point) to commissures X, Y, and Z, respectively. (**D**) ICDs are shown as yellow dotted lines. Cusp angles α, β, and γ correspond to the LCC, RCC, and NCC, respectively. Green arrows indicate the horizontal shift of commissures from the 120° even positions. (**E**) Red circle represents the graft contour. Yellow points X′, Y′, and Z′ indicate predicted commissural positions after aortic root reimplantation. Cusp angles are preserved postoperatively. (**F**–**H**) Sagittal views along the red (**F**), blue (**G**), and green (**H**) cutting planes (from panel **C**), perpendicular to the VBR plane. “D” denotes the difference in radius between the estimated VBR and the commissural circle. Predicted CH is calculated as CH ≈ √(D^2^ + measured CH^2^). CH, commissural height; ICD, intercommissural distance; LCC, left-coronary cusp; NCC, noncoronary cusp; RCC, right-coronary cusp; SVA, sinus of Valsalva aneurysm; VBR, virtual basal ring

To evaluate the feasibility of VSRR, electrocardiogram-gated CT data were analyzed using 2 approaches. First, the 3D shape of the SVA (**Fig. 2A**) guided multiplanar reconstruction of the aortic root to measure the VBR area and perimeter (**Fig. 2B**), commissural circle area (**Fig. 2C**–**2E**), ICDs (**Fig. 2D**), CHs (**Fig. 2F**–**2H**), geometric heights, and effective heights, as previously described,^13,14)^ using the Vitrea image processing system (Canon Medical Systems, Tochigi, Japan). **Table 1** summarizes all CT measurement values. The estimated VBR diameter was 26.9 mm, calculated from the VBR area (566.6 mm^2^) and perimeter (84.3 mm), assuming a circular geometry, using the equations: VBR diameter = 2 × √(VBR area/π), and VBR diameter = VBR perimeter/π. The commissural circle area was 1175.4 mm^2^, corresponding to a diameter of 38.7 mm; thus, the radial difference between the estimated VBR and commissural circle was 5.9 mm. Intercommissural cusp angles for the LCC, RCC, and NCC were calculated from the ICDs (**Fig. 2D**) using the formula: cusp angles (α, β, γ) ≈ 360 × ICDs (XY, YZ, ZX)/(XY + YZ + ZX). Following root reimplantation, each commissure was predicted to move medially and upward into the graft (**Fig. 2E**). Predicted CHs above the VBR plane were derived from the measured CHs using the Pythagorean theorem: predicted CH ≈ √(D^2^ + measured CH^2^), where D represents the radial difference between the estimated VBR and the commissural circle (5.9 mm in this case).^13)^

**Table 1:** Preoperative and postoperative CT measurements and estimated (predicted) values derived from these measurements

Parameter	Preop	Postop
VBR area	566.6 mm^2^	522.9 mm^2^
VBR perimeter	84.3 mm	82.9 mm
Estimated VBR diameter	26.9 mm	26.1 mm
VBR diameter/BSA (>13)	16.8 mm/m^2^	16.3 mm/m^2^
Commissural circle area	1175.4 mm^2^	555.3 mm^2^
Commissural circle diameter	38.7 mm	26.6 mm
Intercommissural distance		
LCC side	29.3 mm	23.7 mm
RCC side	31.2 mm	24.3 mm
NCC side	35.7 mm	25.3 mm
Intercommissural cusp angle		
LCC side	110°	116°
RCC side	117°	119°
NCC side	133°	124°
Measured commissural height (above VBR)		
Between NCC and LCC	16.2 mm	22.2 mm
Between LCC and RCC	6.3 mm	13.1 mm
Between RCC and NCC	19.6 mm	23.9 mm
Predicted commissural height		
Between NCC and LCC	17.3 mm	n/a
Between LCC and RCC	8.7 mm	n/a
Between RCC and NCC	20.5 mm	n/a
Geometric height		
LCC	18.1 mm	18.5 mm
RCC	16.6 mm	18.4 mm
NCC	24.5 mm	22.1 mm
Effective height		
LCC	12.0 mm	10.0 mm
RCC	13.3 mm	9.6 mm
NCC	13.8 mm	10.0 mm
cCL (with a VBR diameter of 25 mm)	Predicted cCL	Measured cCL
LCC	5.6 mm	4.6 mm
RCC	4.1 mm	5.1 mm
NCC	12.0 mm	5.5 mm
Mean coaptation length	7.2 mm	5.1 mm

BSA, body surface area; cCL, cusp coaptation length; LCC, left-coronary cusp; n/a, not applicable; NCC, noncoronary cusp; Preop, preoperative; Postop, postoperative; RCC, right-coronary cusp; VBR, virtual basal ring

**Fig. 3:**
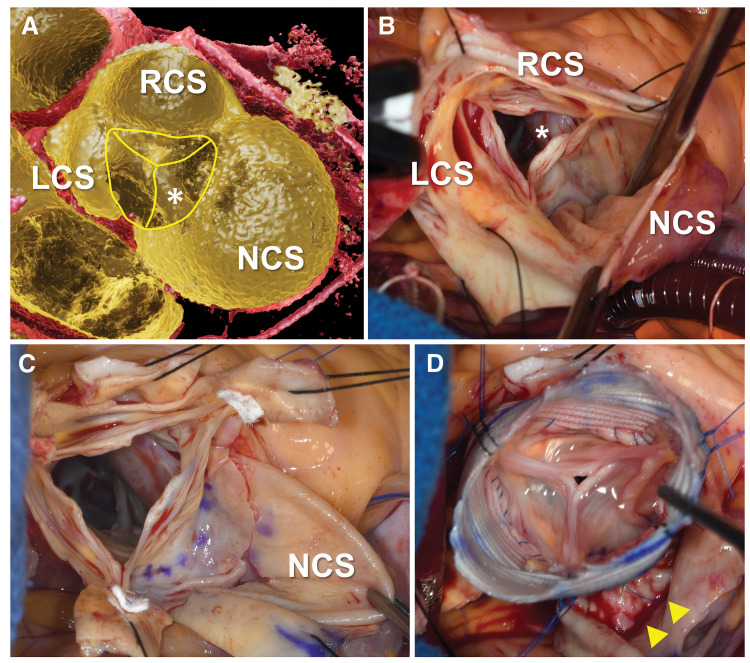
CT-based simulated surgeon’s view and intraoperative photographs. Asterisks indicate the inferior margin of the membranous septum. (**A**) Preoperative 3DCG images simulating the surgeon’s view. (**B**) Intraoperative photograph showing the surgeon’s view of the aortic root. (**C**) The aortic root after resection of the RCS and LCS and incision of the SVA along the annulus of the NCS (dotted blue markings). The SVA wall and the underlying right atrial wall were extremely thin and fragile. (**D**) Spared aortic valve after reimplantation, showing even heights of the cusps’ free margins. Yellow arrowheads indicate running sutures between the remnant SVA wall and graft skirt, reinforcing the incisional portion of the SVA wall. 3DCG, 3D computer graphics; LCS, left-coronary sinus; RCS, right left-coronary sinus; SVA, sinus of Valsalva aneurysm

Second, high-resolution 3DCG images were reconstructed from the CT data using Viewtify software^12)^ (SCIEMENT, Tokyo, Japan). The contours of the annulus and membranous septum, along with the SVA distension, were visualized from a simulated surgeon’s perspective, mimicking intraoperative fields (**Fig. 3A**). Based on these assessments, VSRR was considered feasible for this patient.

Our principal techniques for aortic root reimplantation have been described previously.^15)^ Briefly, we used a large-sized (30–34 mm) Dacron tube graft, which was 6–8 mm larger than the target VBR diameter. The base and top of the graft were plicated to recreate neo-sinuses, thereby maintaining vortex blood flow after root reimplantation.^16)^ For surgical planning in this case, a 32-mm straight graft was selected for reimplantation, with the base plicated to 29 mm, targeting a VBR diameter of 25 mm to account for aortic wall thickness (4 mm). This target VBR diameter was reduced from the preoperative CT measurement (26.9 mm) because the patient had moderate AR, and sufficient cusp coaptation was expected. With this target diameter, an estimated mean cCL ≥6 mm was confirmed using the formula: cCL of each cusp = geometric height of each cusp – target VBR radius, as sufficient for effective coaptation^17,18)^ (**Table 1**). It was also confirmed that the target VBR diameter satisfied the criterion VBR diameter/BSA >13 mm/m^2^ to avoid excessive annular reduction and subsequent aortic valve stenosis^17,19)^ (**Table 1**). Commissural positions were adjusted both horizontally and vertically. Horizontally, cusp angles were mapped onto the graft: a 1-degree match on a 32-mm graft corresponded to 0.28 mm; commissure X (between the NCC and LCC) was shifted 3 mm toward the LCC because the cusp angle of the LCC (110°) was 10° less than 120°. Commissure Z (between the NCC and RCC) was shifted 1 mm toward the RCC, as the cusp angle of the RCC (117°) was 3° less than 120° (**Fig. 2D**). Vertically, predicted CHs (X′, Y′, Z′) above the VBR plane (**Fig. 2F**–**2H**) served as intraoperative references for fixing the commissures to the graft (shown as a red circle in **Fig. 2E**).

The patient underwent aortic root reimplantation via median sternotomy. After establishing cardiopulmonary bypass, a right atriotomy was performed; no perimembranous ventricular septal defect was observed behind the tricuspid valve. Following aortic cross-clamping, the ascending aorta was transected, and crystalloid cardioplegic solution was selectively infused into both coronary arteries to achieve cardiac arrest. Subsequently, retrograde blood cardioplegia was administered every 20 min via the coronary sinus. Coronary buttons were trimmed. The aortic valve was tricuspid, with a prominent NCC facing the SVA (**Fig. 3B**). Intraoperative measurements using Schäfers’ ruler^20)^ demonstrated geometric heights of 17 mm (LCC), 18 mm (RCC), and 25 mm (NCC), consistent with preoperative CT values.

The supravalvular aortic wall of the right and LCSs and the SVA aneurysmal wall were incised, leaving a 5-mm margin above the annulus (**Fig. 3C**). The subvalvular tissue beneath the SVA, including the right atrial wall, was extremely thin and fragile. A 32-mm Hemashield Platinum Woven Double Velour straight graft (Getinge, Gothenburg, Sweden) was selected. Three plication stitches were applied above the graft skirt (5 mm width) to reduce the base diameter to 29 mm. Six 3-0 monofilament mattress sutures with 5-mm pledgets were placed beneath the 3 commissures and nadirs for subvalvular fixation. At the NCC, the remnant SVA wall reinforced the fragile subvalvular tissue. To ensure hemostasis, double running sutures were placed between the graft skirt and remnant SVA wall (**Fig. 3D**).

Commissural positions were finalized under direct inspection with reference to CT-based measurements, confirming even cusp heights and adequate coaptation (**Fig. 3D**). The CH between the RCC and NCC was adjusted from 21 to 20 mm based on a water test (predicted CH: 20.5 mm); no adjustments were required for the other commissures. Supravalvular rims were secured inside the graft using 4-0 monofilament running sutures. No additional cusp repair was necessary. Coronary buttons were reimplanted onto the graft using the Carrel patch technique. Three vertical plication sutures were applied from the midpoints between the commissural tops to the distal end of the graft, reducing the graft diameter to 29 mm and maintaining a reconstructed commissural circle diameter of 25 mm, matching the estimated VBR. The distal aortic anastomosis was completed, and the ascending aorta was declamped.

Weaning from cardiopulmonary bypass was uneventful. The postoperative course was smooth, and the patient was discharged on POD 13. Postoperative CT measurements are shown in **Table 1**. The VBR and commissural circle (STJ) diameters were reduced compared with preoperative values. The cusp angles showed a tendency to maintain a smaller LCC and larger NCC after root reimplantation. The tendency toward a lower CH (Y′ in **Fig. 2E** and **2G**) between the LCC and RCC and a higher CH (Z′ in **Fig. 2E** and **2H**) between the RCC and NCC was also preserved. The mean postoperative cCL was 5.1 mm. Transthoracic echocardiography at discharge showed mild AR with a peak aortic valve velocity of 1.36 m/s. Mild AR remained stable at 1-year follow-up.

## DISCUSSION

Reports of VSRR for SVA repair remain rare. Akashi et al. first described successful VSRR for an unruptured SVA in 2005,^4)^ using a remodeling technique to repair multiple sinus aneurysms in a patient with normal annular size and mild preoperative AR. Subsequent reports have predominantly employed the reimplantation technique for unruptured SVA,^5–7)^ ruptured SVA,^8,9)^ and SVA presenting with acute coronary syndrome.^10)^ A large case series has also documented a small number of cases managed with the reimplantation technique.^11)^

In the present case, the VBR measured 26.9 mm, while the STJ (commissural circle) and ascending aorta were dilated to 38.7 and 44.0 mm, respectively. The AR was attributed to dilation of the sinus of Valsalva and ascending aorta and was classified as type Ia + Ib according to Boodhwani et al.^21)^. This condition could theoretically have been corrected by partial remodeling of the NCS alone. Nevertheless, we elected the reimplantation technique for 2 reasons. First, supravalvular single-line anastomoses carried a high risk of bleeding because the SVA wall media was deficient and replaced by atrophic muscular tissue.^22)^ In contrast, reimplantation secures hemostasis by covering all supravalvular suture lines with a graft. In this case, the fragile subvalvular tissue beneath the SVA necessitated additional sealing sutures between the graft skirt and the aneurysmal wall. Second, all ICDs, including those of the LCCs and RCCs, were distended; the reimplantation technique allows more precise control of commissural positions using CT-based preoperative planning compared with the remodeling technique.

Repairing the unusual anatomical structures of the SVA and associated aortic valve cusps requires meticulous intraoperative inspection within the constraints of limited cardiac arrest time. In our case, the planned commissural positions were highly variable (cusp angles: 110°, 117°, 133°; CHs: 17 mm, 9 mm, 20 mm), making it nearly impossible to determine correct positions by visual inspection alone. Uneven cusp heights during commissural fixation would have necessitated additional cusp repair to ensure adequate coaptation. However, recent evidence suggests that additional cusp repair during VSRR negatively impacts long-term AR recurrence.^23)^ Therefore, achieving VSRR without additional cusp repair is ideal when aortic valve leaflet deformity is minimal, even in cases with disproportionate root anatomy. In this context, a large-sized straight graft may be preferable, as it allows greater flexibility in determining CHs within the graft compared with a pre-shaped neo-sinus graft, particularly in cases with markedly discrepant CHs, such as the present case.^13)^

The reconstructed aortic root in this case was re-evaluated using postoperative CT (**Table 1**). The commissural circle (STJ) diameter was reduced to 26.6 mm, which was nearly equal to the VBR diameter (26.1 mm). The discrepancies in cusp angles and CHs tended to be preserved postoperatively. However, postoperative CHs were 3.4–4.9 mm greater than the preoperatively predicted values, presumably because the commissural positions became vertically distended under aortic pressure within the crimped graft, whereas intraoperative CHs were determined in an unpressurized graft. Regarding aortic valve cusp coaptation, the postoperative cCLs of the LCC and RCC were consistent with the predicted values. However, the predicted cCL of the NCC (12.0 mm) may have been overestimated as a theoretical value; indeed, the measured postoperative cCL of the NCC was 5.5 mm. Nevertheless, these postoperative findings suggest that this markedly abnormal aortic root geometry was successfully reconstructed using CT-based planning, while preserving the interrelationships among the disproportionate cusps and commissural positions.

Recent studies of CT-based aortic root measurements demonstrate that even non-diseased aortic roots exhibit uneven sinus and cusp sizes and variable commissural positions, with the NCS often larger, the CH between the RCC and NCC greater, and the free margin of the RCC longer than the others.^24,25)^ Diseased, dilated aortic roots retain this disproportionality.^14)^ Preoperative, thorough assessment of aortic root geometry—including VBR size, commissural positions, and cusp measurements—facilitates minimally stressful VSRR and ensures secure valve coaptation by reducing uncertain intraoperative adjustments and unnecessary cusp repairs. Moreover, 3D visualization from a surgeon’s perspective aids surgical planning,^12)^ particularly in identifying the annulus, determining the incisional line of the SVA wall, and assessing the feasibility of VSRR.

## CONCLUSIONS

This report demonstrates successful aortic root reimplantation for an unruptured SVA. Preoperative CT-based aortic root visualization and measurements provided accurate virtual images, enabling precise surgical planning and enhancing the surgeon’s confidence in performing valve-sparing procedures for SVAs with unusual anatomy.

## DECLARATIONS

### Availability of data and materials

The datasets supporting the conclusions of this article are included within the article.
